# Treatment Satisfaction, Efficacy, and Safety of Delgocitinib Ointment for Atopic Dermatitis‐Induced Rash on the Face and Neck: Efficacy at Reducing Local Side Effects of Topical Steroid and Tacrolimus Ointment

**DOI:** 10.1111/1346-8138.17817

**Published:** 2025-06-26

**Authors:** Masatoshi Abe, Atsuyuki Igarashi, Hiroyuki Kitajima, Hiroyuki Toyama, Kenji Kabashima, Hidehisa Saeki

**Affiliations:** ^1^ Kojinkai Sapporo Skin Clinic Sapporo Japan; ^2^ Igarashi Dermatology Higashigotanda Tokyo Japan; ^3^ Torii Pharmaceutical Co., Ltd. Tokyo Japan; ^4^ Department of Dermatology Kyoto University Graduate School of Medicine Kyoto Japan; ^5^ Department of Dermatology Nippon Medical School Tokyo Japan

**Keywords:** atopic dermatitis, delgocitinib, face, neck, ointment

## Abstract

This open‐label, single‐arm, multicenter clinical study evaluated the treatment satisfaction, efficacy, and safety of 0.5% delgocitinib ointment twice daily for 12 weeks, applied to face/neck lesions caused by ≥ 3 months use of topical steroids or tacrolimus ointment in patients with atopic dermatitis (AD). The primary endpoint was change in treatment satisfaction (Treatment Satisfaction Questionnaire for Medication‐9 [TSQM‐9]). Secondary endpoints included changes in modified Eczema Area and Severity Index (mEASI) and EASI scores, the numerical rating scale (NRS) for pruritus, the severity of side effects, skin condition at target lesions, disease control (Atopic Dermatitis Control Tool [ADCT]), and preference for delgocitinib ointment (Patient Preference Questionnaire [PPQ]). Overall, 38 patients (mean age 40 years) with a duration of AD of 30.7 years were included in the study. Scores of TSQM‐9 for global satisfaction (Week 0: 58.8 ± 14.7; Week 4: 68.5 ± 16.6; Week 12: 73.2 ± 16.0), effectiveness (57.0 ± 14.1; 67.0 ± 12.6; 70.9 ± 13.4), and convenience (64.9 ± 15.0; 73.6 ± 12.7; 76.1 ± 13.6) (all cases *p* < 0.01), mEASI (Week 0: 1.31; Week 12: 0.68, *p* < 0.001) and NRS (4.53; 2.71, *p* < 0.001) scores, and ADCT (8.3; 5.6, *p* < 0.01) were improved significantly. Compared with that at Week 0, the incidence of ≥ mild skin atrophy, telangiectasia, and skin irritation decreased. The PPQ showed that approximately 80% of patients rated all queries as “strongly agree” or “agree.” In addition, skin conditions were significantly improved for all items (dryness, feel, firmness/elasticity, and appearance) at 1–2 weeks and 12 weeks (*p* < 0.001). The present results demonstrate that delgocitinib ointment might be an effective option for switching treatment for face/neck AD in patients with skin lesions, or for those who are concerned about the development of side effects related to steroids or tacrolimus.

**Trial Registration:** Japan Registry of Clinical Trials (jRCTs031230063)

## 1 Introduction

The goal of treatment for atopic dermatitis (AD) is “to achieve and maintain a state in which there are no symptoms, or if present, they are minor and do not interfere with daily life or require much medication. If this level cannot be reached, the goal is to maintain a state in which symptoms are mild or slight and there is no sudden worsening that interferes with daily life” [1]. To achieve this, patients are treated with drugs, topical therapy/skin care for physiological abnormalities of the skin, and therapies that manage aggravating factors, based on the patient's condition, including the severity of symptoms and patient background. When treating AD, it is critical to quickly and reliably suppress AD inflammation. Topical medications currently under investigation in Japan for this purpose include topical steroids, tacrolimus ointment (topical calcineurin inhibitor), delgocitinib ointment (a Janus kinase [JAK] inhibitor; topical JAK inhibitor), and difamilast ointment (phosphodiesterase 4 [PDE4] inhibitor; topical PDE4 inhibitor). Recently, tapinarof cream, an aryl hydrocarbon receptor agonist, has also been developed and is expected to be a new therapeutic option for AD. The basic treatment strategy involves the selection and combination of these drugs [1, 2]. Topical steroids are often used as the first‐line treatment in the acute phase of AD because they have a rapid onset of action, are highly effective, and can be used at different strengths depending on the severity of the condition [3]. However, they often cause local side effects such as skin atrophy and telangiectasia and, therefore, it is recommended to gradually reduce the dose or switch to a proactive treatment, such as tacrolimus ointment after achieving rapid remission. In addition, it is thought that misunderstandings about topical steroids can lead to fear or aversion to them, which in turn can lead to decreased medication adherence, and the importance of patient education was previously emphasized [1, 2].

Tacrolimus ointment appears to be useful for AD skin lesions in patients who have concerns about topical steroids. It is also expected to be effective at treating facial and neck lesions [1, 2]. However, unlike topical steroids, it has limitations regarding its use: it is prohibited for use on eroded or ulcerated skin, there are dosage restrictions by age, and its efficacy is limited. In addition, although treatment with tacrolimus ointment does not cause skin atrophy as often as that caused by the long‐term use of topical steroids, skin irritation, which is a local side effect, can lead to a decrease in medication adherence [1, 2].

In Japan, the topical JAK inhibitor, delgocitinib ointment, was approved in January 2020 and the PDE4 inhibitor, difamilast ointment, was approved in September 2021 for the treatment of AD. Since the approval of the subcutaneous injectable biologic dupilumab, additional biologics such as nemolizumab, tralokinumab, and lebrikizumab, as well as oral JAK inhibitors (baricitinib, upadacitinib, and abrocitinib) have also been approved. This has led to a significant change in how AD is treated, with high efficacy confirmed for moderate to severe AD that is difficult to treat with topical steroids or anti‐inflammatory topical drugs such as tacrolimus ointment. However, the basic treatment for AD remains topical anti‐inflammatory drugs.

A survey of topical drug use for AD was conducted in 2022, in which physicians reported that delgocitinib ointment has few safety concerns and patients had a good feeling when it was used [3]. However, because it is a relatively new drug, there is not yet sufficient evidence for its use in combination with other drugs or for switching from other drugs. Although topical steroids and tacrolimus ointment have been used to treat patients for many years, a decrease in medication adherence to those ointments was reported because of local side effects and misunderstanding about steroids, which leads to a sense of fear and avoidance of topical steroids [1, 2].

Therefore, in this study, we investigated the usefulness of delgocitinib ointment for facial and neck lesions in patients with AD who had used topical steroids or tacrolimus ointment for ≥ 3 months and had developed or were concerned about side effects. We also investigated the usefulness of switching to delgocitinib ointment and its efficacy in reducing the local side effects of topical steroids and tacrolimus ointment.

## 2 Methods

### 2.1 Study Design

This open‐label, single‐arm, multicenter clinical study consisted of 12 weeks of treatment with delgocitinib ointment in patients with a rash on their face and/or neck related to AD, and was conducted between May and December 2023. This study was reviewed and approved by a certified clinical research ethics committee (certification number: CRB3210001) at each study site, registered with the Japan Registry of Clinical Trials (jRCTs031230063), and carried out in accordance with the principles expressed in the Declaration of Helsinki. All patients provided written informed consent before initiating the study. This study was conducted as a multicenter‐specific clinical research project by the Non‐Profit Organization Health Institute Research of Skin (Chiyoda Ward, Tokyo, Japan) at nine medical institutions where dermatology specialists provided medical care.

### 2.2 Patients

In this study, AD patients aged ≥ 18 years at the time of informed consent, who had used topical steroids or tacrolimus ointment on facial and neck lesions for ≥ 3 months, who had experienced local side effects such as skin atrophy, telangiectasia, and rosacea‐like dermatitis related to the use of topical steroids on the face and/or neck, or who were expected to use topical steroids long‐term and were concerned about the development of local side effects, or patients who had experienced or were concerned about local side effects such as skin irritation and rosacea‐like dermatitis related to the use of tacrolimus ointment on the face and/or neck, and who provided written consent to participate in this study, were included.

Patients who met any of the following exclusion criteria were excluded from the study: patients who were using the strongest topical steroids or delgocitinib ointment on the face or neck; were using difamilast ointment; had obvious erosive lesions on the face and/or neck; were pregnant, might have been pregnant, or wished to become pregnant, or were breastfeeding during the study; were previously treated with delgocitinib ointment; previously received systemic therapy with oral medications (steroids, cyclosporine, JAK inhibitors) or phototherapy such as ultraviolet therapy ≤ 3 months before baseline; had received treatment with biologics (dupilumab, nemolizumab) ≤ 6 months before baseline; had participated in clinical trials or other studies ≤ 6 months before baseline; or were ineligible to participate in the study as determined by the principal investigator or other researchers.

### 2.3 Treatment Protocol

Topical steroids or tacrolimus ointment used on the face and neck of the patients were switched to 0.5% delgocitinib ointment twice daily for 12 weeks according to the approved dosage and administration.

Systemic therapy for AD (oral and injectable medications) was prohibited during the study period. However, the continued use of antihistamines and Kampo medicines indicated for AD that were being used at the start of treatment was permitted, but changes in their dosage and administration during the study period were prohibited.

For the target sites (facial and neck lesions), patients were prohibited from using topical medications other than delgocitinib ointment, and initiating care with moisturizers and changing their dosage and administration. For lesions other than those on the face and neck, only the use of topical steroids or tacrolimus ointment was permitted, and the use of delgocitinib ointment and difamilast ointment was prohibited. In addition, phototherapy such as ultraviolet therapy and other treatments that could affect the efficacy evaluation of the study drug were also prohibited. If acute treatment was required for AD because of the exacerbation of facial and neck lesions or exacerbation of lesions at other sites, patients discontinued the study after recording the evaluation items.

### 2.4 Endpoints and Assessments

The primary endpoint was a change in treatment satisfaction assessed using Treatment Satisfaction Questionnaire for Medication‐9 (TSQM‐9) scores before and after switching from topical steroids or tacrolimus ointment to delgocitinib ointment at Weeks 0, 4, and 12.

Secondary endpoints were changes in the modified Eczema Area and Severity Index (mEASI) and EASI scores, and numerical rating scale (NRS) of pruritus on the face and neck at Weeks 0, 1–2, 4, 8, and 12; changes in the severity of side effects at target lesions (skin atrophy, telangiectasia, rosacea‐like dermatitis) at Weeks 0, 1–2, 4, 8, and 12; changes in skin conditions at target lesions (dryness, feel, firmness/elasticity, appearance) at Weeks 0, 1–2, and 12; changes in disease control (Atopic Dermatitis Control Tool [ADCT]) at Weeks 0, 4, 8, and 12; and changes in preference for delgocitinib ointment (Patient Preference Questionnaire [PPQ]) at Weeks 4 and 12.

At the time of registration, the following background information was collected: sex, age, medical history, complications, duration of AD, and concomitant medications. In addition, local side effects related to topical steroid use (skin atrophy, telangiectasia, rosacea‐like dermatitis) and tacrolimus ointment (skin irritation, pruritus, rosacea‐like dermatitis) were evaluated on a 5‐point scale: 1: none (or concern about development), 2: mild (signs observed), 3: moderate, 4: moderate, and 5: severe.

The following evaluations were performed at the start of treatment with delgocitinib ointment (Week 0), 1–2, 4, 8, and 12 weeks after the start of treatment, or at discontinuation (final evaluation). The TSQM‐9 was used to assess patient satisfaction with delgocitinib ointment. The mEASI score for the face and neck and the EASI score were used to assess the severity of AD. Pruritus on the face and neck was evaluated using the NRS. The ADCT was used to assess the disease control status of AD. A 4‐item PPQ on a 4‐point scale (0: not at all, 1: no, 2: yes, 3: strongly agree) was used to assess patient preference for the treatment with delgocitinib ointment based on the following five items: current treatment for my face and neck is more effective than the previous treatment; current treatment for my face and neck is easier to use than the previous treatment; current treatment for my face and neck has fewer side effects than the previous treatment; I find the current treatment for my face and neck more acceptable than the previous treatment; and I prefer the current treatment for my face and neck to the previous treatment. In addition, as a patient questionnaire on skin symptoms, patients were asked to answer the following four questions about the feeling of using the study drug at the application site on an 11‐point scale (0 to 10): dryness (0 points: dry to 10 points: moist), feel (0 points: rough to 10 points: smooth), firmness/elasticity (0 points: no firmness/elasticity to 10 points: firm/elastic), and appearance (0 points: bad appearance to 10 points: good appearance). Medication adherence to delgocitinib ointment was also evaluated at each visit.

As a safety evaluation item, all adverse events were recorded, and the causal relationship between this study and the study drug was evaluated throughout the study period.

### 2.5 Statistical Analysis

The sample size was estimated at approximately 40–100 patients when the change in “Global satisfaction” of TSQM‐9, the primary endpoint after switching to delgocitinib ointment, was set to 5–9 with a power of 0.8, a significance level of 0.05, and a dropout rate of 20%. Considering the capability of study sites to recruit patients during the registration period, the target sample size was set at 40 patients.

The efficacy analyses were carried out using the Full Analysis Set, which included all patients who received at least one dose of the study treatment and had any subsequent efficacy observation. Safety was evaluated in the Safety Analysis Set, which included all patients who received the study drug at least once after registration and had at least one safety data point.

For the analysis, summary statistics and the mean ± standard deviation (SD) were calculated for the transition and change in scores. The internal consistency reliability of the TSQM‐9 subscales (Effectiveness, Convenience, and Global Satisfaction) was assessed using Cronbach's alpha coefficient at each scheduled visit. Paired *t*‐tests were used to compare results at pre‐treatment and each observation time point, with a two‐sided significance level of 0.05. In principle, if data were missing after the start of the study, the last observation carried forward principle was applied, including discontinued patients, and the last data obtained after the start of the study were used for imputation in the evaluation of changes over time. However, for evaluation items used to calculate proportions, if data required for calculating the exact value were missing after the start of the study, imputation was not performed, and the patient was excluded from the denominator for the calculation. No imputation was performed for other missing values. JMP Ver.17 (JMP Statistical Discovery LLC., Cary, NC, USA) was used for statistical analyses.

## 3 Results

### 3.1 Baseline Characteristics of the Patients

Of 40 patients enrolled in the study, 38 (22 males and 16 females) were included in the analysis, excluding 2 males who did not visit the hospital after registration. The mean ± SD age of patients was 40.3 ± 12.4 years (40.9 ± 11.9 for males and 39.4 ± 13.5 for females), and the mean duration of AD was 30.7 ± 13.3 years (Table 1). The mean mEASI score for the face and neck was 1.31 ± 0.88, and the mean EASI score was 8.90 ± 8.32. No sex differences were observed for any baseline characteristics.

**TABLE 1:** Baseline characteristics of patients with AD.

	Total (*n* = 38)	Male (*n* = 22)	Female (*n* = 16)
Age, years (mean ± SD)	40.3 ± 12.4	40.9 ± 11.9	39.4 ± 13.5
Range (min–max)	(22–64)	(22–63)	(22–64)
Duration of AD, years (mean ± SD)	30.7 ± 13.3	31.8 ± 11.5	29.3 ± 15.7
Allergic diseases, *n*	3	2	1
Asthma	1	0	1
Pollen allergy	1	1	0
Allergic retinopathy	1	1	0
*AD severity score at Week 0*			
Face and neck			
mEASI (mean ± SD)	1.31 ± 0.88	1.50 ± 1.05	1.05 ± 0.51
Pruritus NRS (mean ± SD)	4.53 ± 2.10	3.91 ± 1.77	5.38 ± 2.28
Systemic			
EASI (mean ± SD)	8.90 ± 8.32	9.71 ± 9.40	7.79 ± 6.67

Abbreviations: AD, atopic dermatitis; EASI, Eczema Area and Severity Index; mEASI, modified EASI; NRS, numerical rating scale; SD, standard deviation.

Regarding topical anti‐inflammatory drug use on the face and neck at Week 0, 24 patients (63.2%) were using topical steroids, 20 patients (52.6%) were using tacrolimus ointment, and 6 patients (15.8%) were using both. Twenty‐five patients (65.8%) were concomitantly using moisturizers, and 24 patients (63.2%) were on oral antihistamines. The class of steroids at Week 0 was: weak in four patients, medium in 13 patients, strong in two patients, and very strong in five patients. For tacrolimus ointment, five patients used the 0.03% formulation and 15 patients used the 0.1% formulation. Local side effects were observed in 15 out of 24 patients (62.5%), and the incidence rate of local side effects was higher in those using lower‐ranked steroids. The breakdown of local side effects was as follows: skin atrophy in 11 patients, telangiectasia in 13 patients, and rosacea‐like dermatitis in 10 patients. Local side effects were observed in 9 out of 20 patients (45.0%) using tacrolimus ointment, with the following breakdown: skin atrophy in five patients, pruritus in seven patients, and rosacea‐like dermatitis in three patients. No difference was observed in the incidence and severity of local side effects compared with those with the 0.1% formulation (Table 2). All 38 patients (100.0%) were compliant with the treatment using delgocitinib ointment.

**TABLE 2:** Number and incidence rate of patients with local side effects by treatment and strength at week 0.

	Number of patients, *n*	Patients with local side effects, *n* (%)
Topical steroid	24	15 (62.5)
Weak	4	4 (100.0)
Medium	13	10 (76.9)
Strong	2	1 (50.0)
Very strong	5	0
Strongest	0	0
Tacrolimus ointment	20	9 (45.0)
0.03% formulation	5	2 (40.0)
0.1% formulation	15	7 (46.7)

### 3.2 Patient‐Rated Treatment Satisfaction With Delgocitinib Ointment at the Target Lesions

**FIGURE 1:**
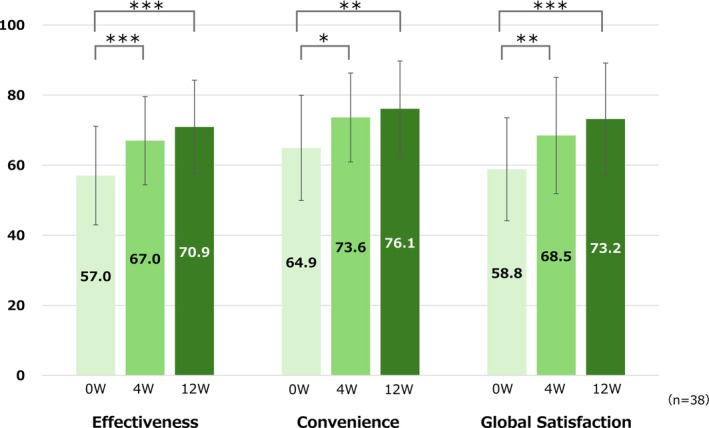
Trends in TSQM‐9 regarding face and neck rash in patients with AD from Week 0 to 12. Values are the mean ± SD. *p < 0.05; **p < 0.01; ***p < 0.001 (vs. week 0, paired *t*‐test). AD, atopic dermatitis; SD, standard deviation; TSQM‐9, treatment satisfaction questionnaire for Medication‐9.

As shown in Figure 1, significant increases were observed in global satisfaction (58.8 ± 14.7 at Week 0, 68.5 ± 16.6 at Week 4, *p* < 0.01, and 73.2 ± 16.0 at Week 12, *p* < 0.001), satisfaction with effectiveness (57.0 ± 14.1, 67.0 ± 12.6, *p* < 0.001, and 70.9 ± 13.4, *p* < 0.001, respectively), and satisfaction with convenience (64.9 ± 15.0, 73.6 ± 12.7, *p* < 0.05, and 76.1 ± 13.6, *p* < 0.01) at 4 and 12 weeks after the start of treatment compared with those at Week 0, indicating an improvement in treatment satisfaction. The internal consistency reliability of the TSQM‐9 subscales, which supports score interpretation, was assessed using Cronbach's alpha coefficient at each time point, as shown in Table 3. At baseline (0W), Cronbach's alpha values for the Effectiveness, Convenience, and Global Satisfaction subscales were 0.57, 0.67, and 0.23, respectively. By Week 4, these values increased to 0.69, 0.91, and 0.65, and at Week 12, to 0.79, 0.90, and 0.65, respectively. Internal consistency for the Convenience and Effectiveness subscales generally improved over the study period, reaching acceptable levels (≥ 0.70) by 4 or 12 weeks. Although the Global Satisfaction subscale demonstrated low internal consistency at 0W, it showed improvement over time, though the values remained below 0.70.

**TABLE 3:** Cronbach's alpha coefficients for Treatment Satisfaction Questionnaire for Medication‐9 (TSQM‐9) subscales.

Subscale	0 weeks	4 weeks	12 weeks
Effectiveness	0.57	0.69	0.79
Convenience	0.67	0.91	0.90
Global satisfaction	0.23	0.65	0.65

### 3.3 Efficacy of Treatment With Delgocitinib Ointment at the Target Lesions

**FIGURE 2:**
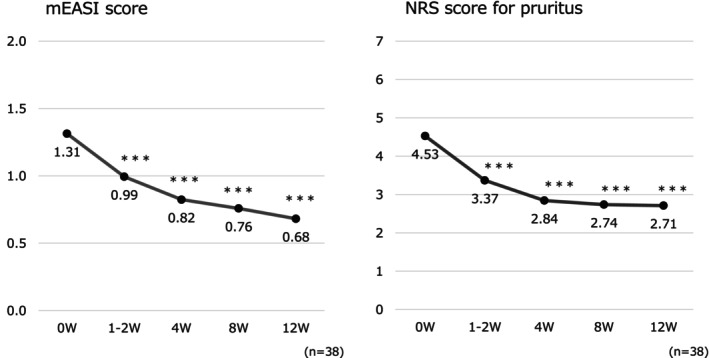
Changes in symptoms on the face and neck over time. ***p < 0.001 (vs. Week 0, paired *t*‐test). mEASI, modified Eczema Area and Severity Index; NRS, numerical rating scale.

A significant decrease in the mEASI scores for the face and neck of patients was found at 1–2, 4, and 12 weeks (*p* < 0.001 at all time points). Similarly, the NRS score for pruritus showed a significant decrease at all observation time points compared with the week 0 scores (*p* < 0.001 at all time points) (Figure 2). The EASI scores also showed improvement (8.90 ± 8.32 at Week 0, 6.12 ± 6.83 at Week 12, *p* < 0.001 at all time points).

**FIGURE 3:**
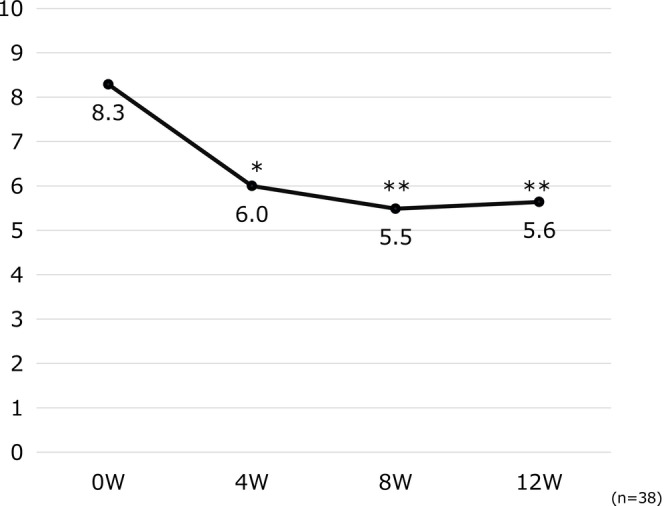
Changes in ADCT over time. *p < 0.05, **p < 0.01 (vs. Week 0, paired *t*‐test). ADCT, atopic dermatitis control tool.

The mean ADCT score, indicating the disease control status, at Week 0 was 8.3, which decreased significantly to 6.0 at 4 weeks (*p* < 0.05) and 5.5 at 8 weeks (*p* < 0.01), and a good disease control status was maintained until the end of the observation period at 12 weeks (5.6, *p* < 0.01) (Figure 3).

**FIGURE 4:**
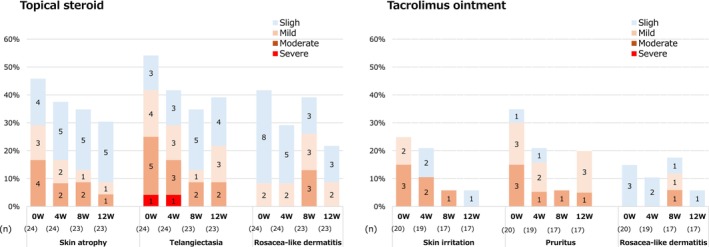
Trends in the severity of local side effects at Week 0.

Local side effects caused by topical steroids at Week 0 were observed in 11 patients (45.8%) with skin atrophy, 13 patients (54.2%) with telangiectasia, and 10 patients (41.7%) with rosacea‐like dermatitis. Of these, mild or greater side effects were observed in 29.2% of patients with skin atrophy, 41.7% with telangiectasia, and 8.3% with rosacea‐like dermatitis. Compared with that at week 0, the proportion of patients with mild or greater skin atrophy and telangiectasia decreased, indicating an improvement. Local side effects related to tacrolimus ointment were observed in five patients (25.0%) with skin irritation, seven patients (35.0%) with pruritus, and three patients (15.0%) with rosacea‐like dermatitis. Mild or greater side effects were observed in 25.0% of patients with skin irritation, 30.0% with pruritus, and 0% with rosacea‐like dermatitis. Compared with that at Week 0, the proportion of patients with mild or greater skin irritation decreased, showing an improvement over time, but no clear decrease was observed for pruritus or rosacea‐like dermatitis (Figure 4).

### 3.4 Assessment of Treatment Satisfaction and Skin Conditions With Delgocitinib Ointment

**FIGURE 5:**
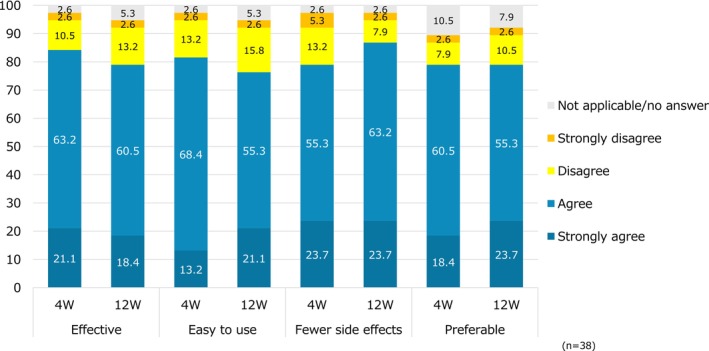
Evaluation of patient preference for therapeutic drugs (PPQ). *Imputation was performed with the last observation carried forward principle for patients whose disease was worsened or who discontinued treatment. PPQ, Patient Preference Questionnaire.

**FIGURE 6:**
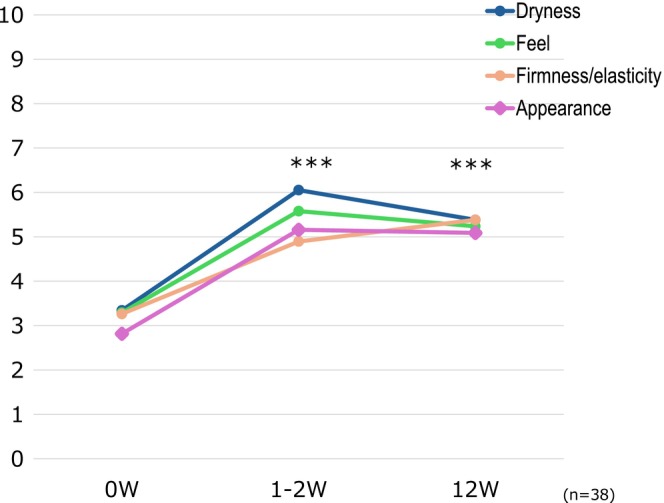
Evaluation of skin condition (questionnaire). ***p < 0.001 (vs. Week 0, Wilcoxon signed‐rank test).

In the PPQ comparing the study drug to the previous topical medication, 84.3% of patients at 4 weeks and 78.9% at 12 weeks answered “strongly agree” or “agree” to the question “Is it more effective than the previous medication?” To the question “Is it easier to use?”, 81.6% at 4 weeks and 76.4% at 12 weeks answered “strongly agree” or “agree”. To the question “Does it have fewer side effects?”, 79.0% at 4 weeks and 86.9% at 12 weeks answered “strongly agree” or “agree”. To the question “Do you prefer it?”, 78.9% at 4 weeks and 79.0% at 12 weeks answered “strongly agree” or “agree” (Figure 5). In addition, the evaluation of skin condition by patients showed a significant improvement in all items (dryness, feel, firmness/elasticity, and appearance) (*p* < 0.001 in all cases) at 1–2 weeks and 12 weeks compared with that at Week 0 (Figure 6).

### 3.5 Discontinued Patients Because of AD Exacerbation

Five patients discontinued the study because of the exacerbation of facial and neck lesions or lesions at other sites (four patients with an exacerbation of facial and neck lesions, and one patient with an exacerbation of lesions at other sites). No clear trends were observed in background characteristics, previous treatment, or severity of AD among these five patients (Table 4).

**TABLE 4:** List of patients who discontinued treatment because of worsened symptoms of AD.

No	Exacerbated site	Timing (weeks after switch)	Sex	Age (years)	Duration of AD (years)	Previous treatment	Local side effects at the time of enrollment	Severity of AD at the time of enrollment		Comorbidity
								mEASI on face/neck	EASI score	
1	Exacerbation of rash outside the face/neck	2 weeks	Male	41	40	Tacrolimus ointment (0.1% formulation)	None	0.4	11.4	Herpes simplex Verruca vulgaris
2	Exacerbation of rash at the target lesion	4 weeks	Female	22	21	Tacrolimus ointment (0.1% formulation)	None	0.8	8.2	Verruca
3	Exacerbation of rash at the target lesion	4 weeks	Female	49	44	Topical steroid (weak)	Skin atrophy Telangiectasia Rosacea‐like dermatitis	1.4	12.1	None
4	Exacerbation of rash at the target lesion	4 weeks	Male	53	47	Tacrolimus ointment (0.1% formulation)	Skin irritation Pruritus	1.5	9.7	Hyperuricemia Hypertension Constipation
5	Exacerbation of rash at the target lesion	8 weeks	Female	32	32	Tacrolimus ointment (0.03% formulation)	Skin irritation Pruritus	0.9	3.7	Acne vulgaris

Abbreviations: AD, atopic dermatitis; EASI, Eczema Area and Severity Index; mEASI, modified EASI.

### 3.6 Safety

One patient experienced the study‐related aggravation of acne vulgaris 8 weeks after the start of treatment. However, it was mild and did not affect their continuation of the study.

## 4 Discussion

This study investigated treatment satisfaction, efficacy, and safety of delgocitinib ointment in patients with facial and neck AD lesions who had been using topical steroids or tacrolimus ointment for ≥ 3 months. We confirmed an improvement in treatment satisfaction (TSQM‐9), decreases in the mEASI score and NRS score for pruritus, and reductions in the incidence of local side effects such as skin atrophy and telangiectasia related to topical steroid use by switching the treatment to delgocitinib ointment. These results indicate that delgocitinib ointment might be an effective treatment option when switching treatment for face and neck AD in patients with skin atrophy, telangiectasia, and/or rosacea‐like dermatitis caused by the long‐term use of topical steroids or tacrolimus ointment, or in those who are concerned about the development of such adverse events.

Delgocitinib ointment is a small molecule (molecular weight 310.35 g/mol) pan‐JAK inhibitor that inhibits all JAK family kinases (JAK1, JAK2, JAK3, and tyrosine kinase 2) and reduces inflammation associated with AD by suppressing the activation of immune cells [4]. Delgocitinib ointment 0.5% significantly improved skin rash scores, and its acute and 52‐week long‐term safety and efficacy in adult patients with mild to severe AD in clinical trials has been reported [5, 6, 7].

AD in patients in this study appeared to be insufficiently controlled based on the findings that these patients had a mean EASI score of 8.90, mEASI score of 1.31, and NRS score of 4.53 at Week 0, which are indicative of moderate AD symptoms, despite the fact that they were treated with topical steroids or tacrolimus ointment for ≥ 3 months. Such lack of sufficient AD control might be caused by decreased medication adherence to topical steroids or tacrolimus ointment as evidenced by previous studies demonstrating patients' concerns about topical steroids and skin irritation (burning or stinging sensation) caused by tacrolimus ointment, and decreased medication adherence leading to the discontinuation of treatment; therefore, treatment efficacy with these ointments is unlikely to be complete [8, 9].

It has been shown that delgocitinib ointment had sustained efficacy by twice daily applications, and it did not cause local side effects such as skin atrophy, telangiectasia, or irritation (burning or stinging sensations) to the same degree as steroids and tacrolimus [6, 10]. Therefore, in this study, patients were likely to have improved medication adherence to the treatment compared with that with topical steroids or tacrolimus ointment. Our results demonstrated a significant improvement in treatment satisfaction (TSQM‐9) by switching to treatment with delgocitinib ointment. These results suggest that delgocitinib ointment improved not only the physical symptoms of patients but also their mental aspect, leading to increased medication adherence because the treatment satisfaction improved effectiveness, convenience, and global satisfaction. It is conceivable that the formulation characteristics of delgocitinib ointment contributed the improvement in TSQM‐9 convenience scores. In the patient preference evaluation, a high proportion of positive responses was recorded for the item “Is it easier to use than the previous medication?” (Figure 5), suggesting enhanced convenience. Furthermore, patient evaluations of skin condition at the application site indicated improvement in the “feel” item (Figure 6), implying that the favorable tactile sensation of the ointment may have contributed to satisfaction with its ease of use. These findings suggest that the user‐friendliness of delgocitinib ointment may play a role in improving patient satisfaction during treatment transitions from previous topical treatments. However, in the management of facial AD, it is essential to consider additional factors beyond convenience to ensure effective and comprehensive care. Because facial skin is thin and highly visible, the balance between effectiveness and safety, as well as cosmetic outcomes, is critical when treating AD. Topical steroids have strong anti‐inflammatory effects and are effective at suppressing acute inflammation. However, their long‐term use on the face increases the risk of local side effects such as skin atrophy, telangiectasia, and rosacea‐like dermatitis [1, 2]. Although tacrolimus ointment has a lower risk for the development of local side effects compared with topical steroids, it takes a long time to be effective, and it may cause irritation and/or itching [1, 2]. Although not direct comparisons, previous meta‐analyses have demonstrated that delgocitinib ointment has an efficacy equivalent to that of topical steroids, and has a low risk for developing local side effects [11, 12]. In addition, it is expected to be a topical treatment option for the face on the basis of its convenient application.

In the management of moderate to severe AD, recent reports have noted cases of head and neck erythema occurring while dupilumab treatment, for which established treatment options are currently lacking [13]. The efficacy of delgocitinib ointment for this type of erythema has been suggested in several case reports [14, 15]. Although this study did not specifically target dupilumab‐induced head and neck erythema, the demonstrated efficacy and safety of delgocitinib ointment for head and neck AD lesions indicate its potential as a treatment option in this context. However, the data from this study alone are insufficient to draw definitive conclusions, and further clinical investigation is warranted.

Regarding the safety profile of delgocitinib ointment, it is associated with a risk of infection because it inhibits the JAK–STAT signaling cascade. Previous studies reported that infection‐related adverse events were limited to local acne, folliculitis, herpes simplex, and Kaposi's varicelliform eruption, which were resolved by time or appropriate treatments [5, 6, 7].

As previously reported for oral JAK inhibitors, the risk for malignant tumors may be a concerning systemic effect of delgocitinib ointment, which is easily absorbed through the skin because of its low molecular weight [16]. At the time of its approval, the dosage was limited to 5 g twice daily because the maximum dose per application investigated in the clinical trials was 5 g [5, 6, 7]. In a previous clinical study, in which AD patients were treated with delgocitinib 0.5% ointment twice daily (maximum dose per application, 5 g), delgocitinib plasma concentrations were undetectable in many patients during the study period (83.5%–91.1%) [5]. No obvious difference in the proportion of patients with detectable delgocitinib plasma concentrations related to the timing of visits was found. Maximum delgocitinib plasma concentrations at each visit ranged from 4.3 to 11.4 ng/mL. These results indicate that the delgocitinib plasma concentrations in patients with detectable delgocitinib fluctuated around the lower limit of detectable levels and, therefore, its transfer to the blood was negligible.

Such dosage restrictions of up to 5 g may not be sufficient when applying it to a large area of skin. However, if a rash is widespread during the acute phase, a possible treatment strategy might be to quickly reduce the area of the rash using topical steroids, and then switch to delgocitinib ointment. However, it appears that 5 g per application has sufficient efficacy for facial and neck lesions; indeed, the present study, in which a rash on the face and neck was investigated, demonstrated favorable treatment efficacy by switching the treatment to delgocitinib ointment.

In addition, patients treated with weak topical steroids in this study had a high incidence rate of adverse events (4/4 patients, 100%), suggesting that even weak topical steroids cause adverse events related to the cumulative effects of their long‐term use or their application to thin skin areas like the face and neck. The present results demonstrate that delgocitinib ointment might be an effective treatment option when switching treatment for face/neck AD in patients with skin lesions, or in those who are concerned about the development of such side effects.

Although this study demonstrated the efficacy and safety of switching to delgocitinib ointment, several limitations should be noted. First, the sample size was relatively small (*n* = 38 for efficacy analysis), and the observation period was limited to 12 weeks. Furthermore, although Cronbach's alpha values were calculated to assess the internal consistency reliability of the TSQM‐9 subscales, the precision of these estimates may have been affected by the limited sample size, and caution is warranted when generalizing the reported alpha values. Moreover, detailed correlation analyses between TSQM‐9 scores and clinical severity measures (mEASI, EASI, NRS) were not performed, nor were multivariate analyses conducted to identify specific items associated with disease severity. While the primary objective was to evaluate overall satisfaction, efficacy, and safety over time, a better understanding of the relationship between specific aspects of treatment satisfaction and clinical severity could provide valuable insights. Therefore, these findings should be validated through large‐scale studies and/or longer observation periods, and future studies should explore the association between patient satisfaction and disease severity in greater depth.

In conclusion, this study examined switching treatment from topical steroids or tacrolimus ointment to delgocitinib ointment for face and neck rash. The results showed that delgocitinib ointment was an effective and safe option for patients with face/neck AD who are concerned about the side effects of topical steroids or tacrolimus ointment. Furthermore, this study examined switching treatment from topical steroids or tacrolimus ointment to delgocitinib ointment for face and neck rash. In the future, the efficacy and safety of the concomitant use of delgocitinib ointment with other treatments, and its role in proactive therapy, need to be investigated.

## Ethics Statement

Approval of the research protocol by an Institutional Review Board: This study was reviewed and approved by a certified clinical research ethics committee (certification number: CRB3210001).

## Consent

All participants provided written informed consent prior to study enrollment.

## Conflicts of Interest

Masatoshi Abe has received research grants, consulting fees, speaker fees, and/or participated in clinical trials for Amgen, Maruho, AbbVie, Kyowa Kirin, Torii, LEO Pharma, Eli Lilly, Bristol Myers Squibb, Novartis, Sun Pharma, Sanofi, and UCB Pharma. Atsuyuki Igarashi has received advisory board honoraria, consulting fees, or speaker honoraria from AbbVie, Eli Lilly, Japan Tobacco, Maruho, Novartis, Sanofi, LEO Pharma, and Torii Pharmaceutical; he has also received research grants from AbbVie, Eli Lilly, Japan Tobacco, Novartis, Otsuka Pharmaceutical, Amgen, and Sanofi. Kenji Kabashima has received consulting fees, honoraria, grant support, and/or lecture fees from AbbVie, Amgen, Eli Lilly, Kyowa Kirin, Japan Tobacco, LEO Pharma, Maruho, Mitsubishi Tanabe, Ono Pharmaceutical, Pfizer, Procter & Gamble, Sanofi, Regeneron, Taiho, and Torii Pharmaceutical. Hiroyuki Toyama has received speaker's fees from AbbVie, Sanofi, Bristol‐Myers Squibb, Eli Lilly, Maruho, Nippon Boehringer Ingelheim, Taiho Pharmaceutical, Otsuka Pharmaceutical, Pfizer Japan, Torii Pharmaceutical, UCBC Japan, Amgen, Mitsubishi Tanabe Pharma, LEO Pharma, and Novartis; research grants (clinical trials) from AbbVie and LEO Pharma; scholarships from Sun Pharma Japan, Maruho, Taiho Pharmaceutical, and Torii Pharmaceutical; and is an Editorial Board member of The Journal of Dermatology and a co‐author of this article. To minimize bias, he was excluded from all editorial decision‐making related to the acceptance of this article for publication. Hiroyuki Kitajima and Hiroyuki Toyama are employees of Torii Pharmaceutical Co. Ltd.

## Data Availability

The data underlying this article cannot be shared publicly to protect the privacy of individuals who participated in the study. The data will be shared upon reasonable request from the corresponding author.
